# Supplementary material for: Rapid detection and recognition of whole brain activity in a freely behaving Caenorhabditis elegans
Source: PLoS Comput Biol. 2022 Oct 10;18(10):e1010594. doi: 10.1371/journal.pcbi.1010594 (PMC9584436; doi:10.1371/journal.pcbi.1010594)
Supplement: S1 Table — For a given training dataset, identical hyperparameters are used for feature engineering and network training. Neuronal regions (Region) or objects (Object) are used to build KNN and density feature vector, respectively. k and q are parameters for the KNN and neuronal density feature, respectively. d is the dimensionality of the feature embedding space and s is the hypersphere radius. m1, m2 and m3 are margin penalty coefficients. N is the number of neurons to be recognized. (PDF) [file pcbi.1010594.s001.pdf]

Method	Training Dataset	KNN type	Density type	k	q	s	d	m_1	m_2	m_3	N	Usage
CeNDeR	CeNDeR C1	Object	Region	25	20	32	56	1.05	0.00	0.05	163 + 1	M1, M2
CeNDeR	NeRVE	Object	Object	25	10	32	56	1.05	0.00	0.00	73 + 1	M3
