# Supplementary material for: Rapid detection and recognition of whole brain activity in a freely behaving Caenorhabditis elegans
Source: PLoS Comput Biol. 2022 Oct 10;18(10):e1010594. doi: 10.1371/journal.pcbi.1010594 (PMC9584436; doi:10.1371/journal.pcbi.1010594)
Supplement: S2 Table — The recognition model was trained on C1 by using KNN and neuronal density as a concatenated input vector and tested on various benchmarks, from which the mean accuracy was calculated. 4 different combinations (neuronal regions or objects) were used to construct input feature vectors during the training stage, and we refer to M1 in S1 Table for the hyperparameters. If the test benchmark does not contain neuronal region information, we constructed feature vectors using only neuronal objects. (PDF) [file pcbi.1010594.s002.pdf]

KNN	Density	C1 (%)	C2/C3 (%)	NeRVE (%)	NeuroPAL Yu (%)	NeuroPAL Chaudhary (%)
Object	Region	95.48	66.70	50.08	27.79 ± 1.11	42.97 ± 2.35
Object	Object	95.12	61.22	50.44	26.25 ± 3.57	40.47 ± 2.64
Region	Region	95.59	60.54	49.69	25.23 ± 4.98	37.75 ± 3.75
Region	Object	95.00	54.29	49.32	24.82 ± 4.90	36.39 ± 3.15
