# Supplementary figures and images for: Rapid detection and recognition of whole brain activity in a freely behaving Caenorhabditis elegans
Source: PLoS Comput Biol. 2022 Oct 10;18(10):e1010594. doi: 10.1371/journal.pcbi.1010594 (PMC9584436; doi:10.1371/journal.pcbi.1010594)

median: 9.0  
mean: 9.2  
std: 1.1

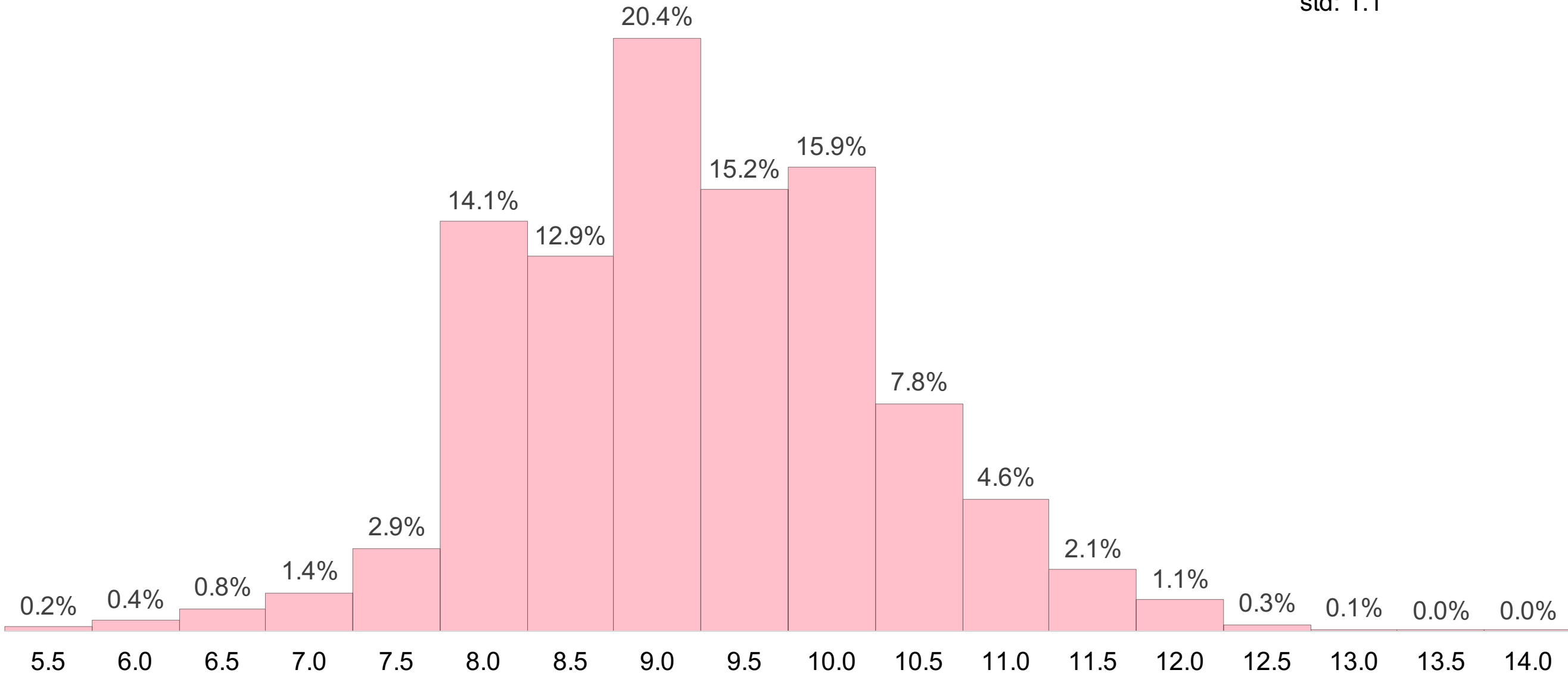

Supplement: S1 Fig — The median and average size is 9, so we design two types of anchor boxes: size 9 and size 7, 11. The second type would better cover regional size ≤ 6 or ≥ 13. See Table 2 for task performance. (PDF) [file pcbi.1010594.s003.pdf]

**A**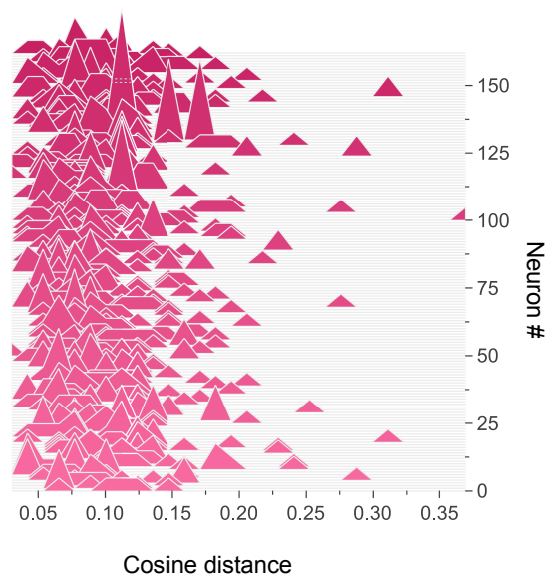**B**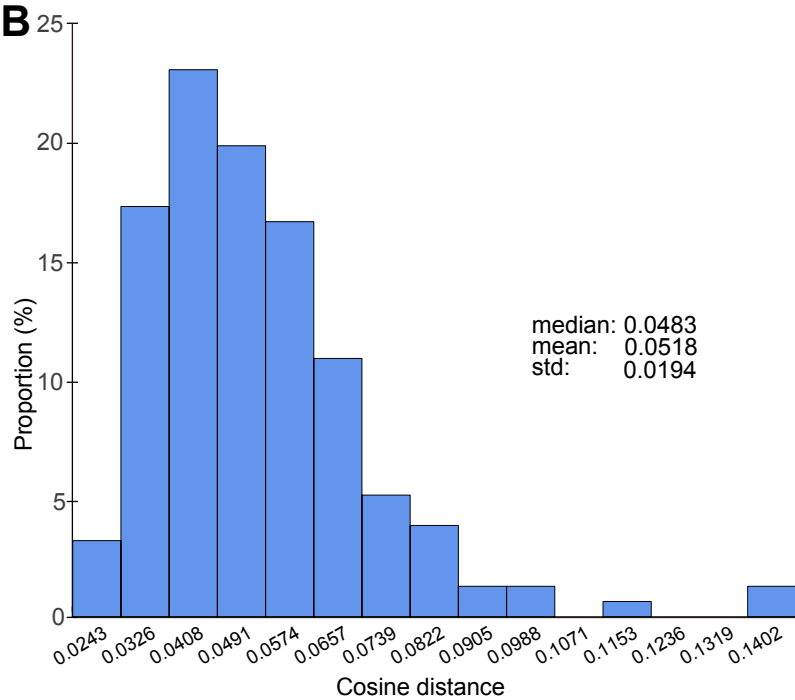**C**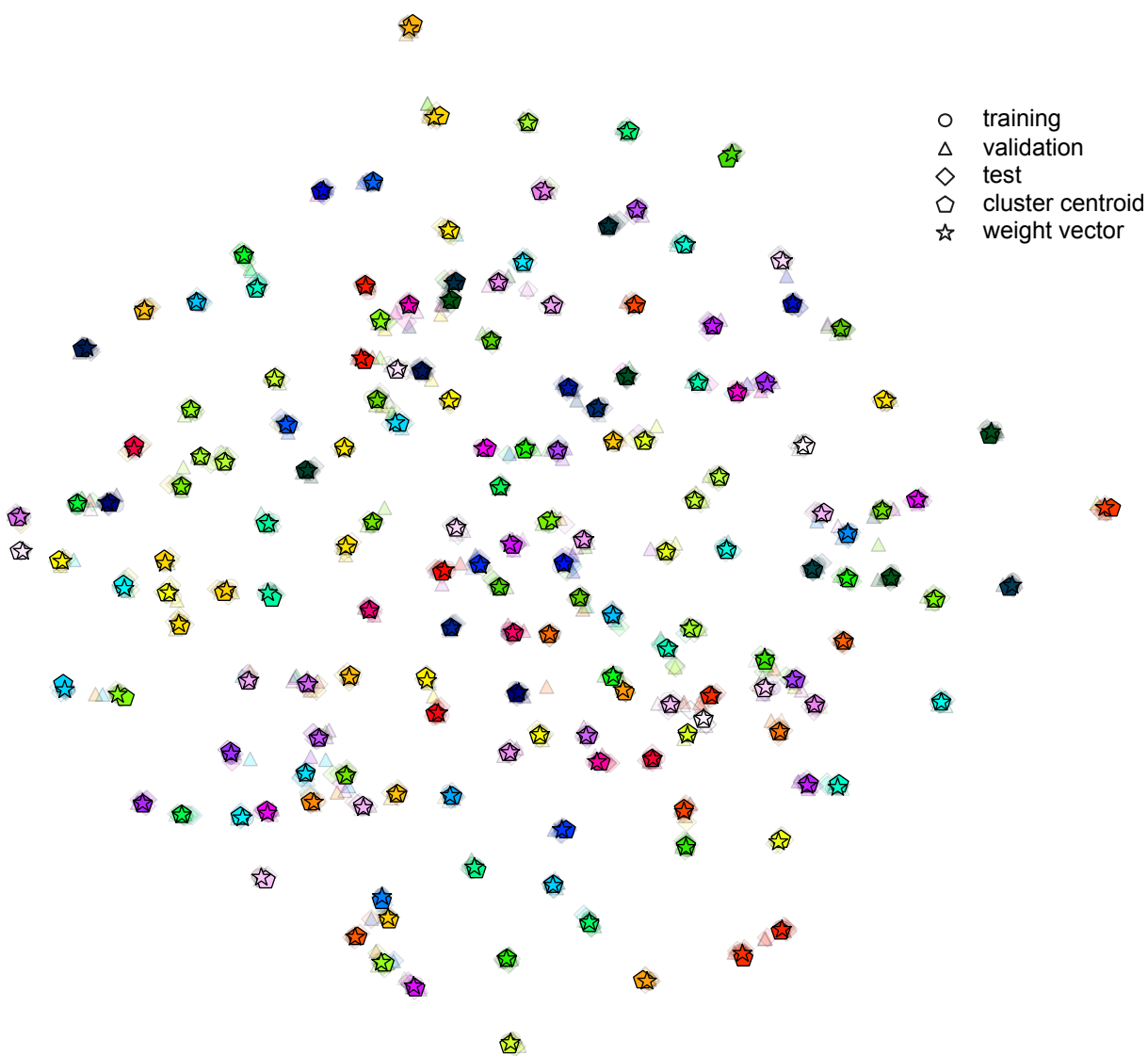

Supplement: S2 Fig — (A) Intra-class cosine distance distributions in every neuron cluster. Y-axis is cluster index; X-axis is the cosine distance between an embedding feature vector c and the corresponding weight vector wξ across the training set. A dashed line indicates a truncation to fit the figure panel into the page. (B) Distribution of cosine distance between a neuron cluster centroid and the corresponding weight vector wξ across the training set. Because the distance is very small (with a median ≈ 0.04), {wj} could represent neuron cluster centers. (C) t-SNE visualization of neuron cluster centroids and weight vectors in the embedding space, related to B. (PDF) [file pcbi.1010594.s004.pdf]
