# Supplementary material for: Rapid detection and recognition of whole brain activity in a freely behaving Caenorhabditis elegans
Source: PLoS Comput Biol. 2022 Oct 10;18(10):e1010594. doi: 10.1371/journal.pcbi.1010594 (PMC9584436; doi:10.1371/journal.pcbi.1010594)
Supplement: S3 Fig — Detection (pink rectangle) and neuron tracking results (blue number inside a pink rectangle) of C2. Note that a raw imaging volume (1024 × 1024 × 18) has been automatically cropped into a smaller size (273 × 237 × 18) to embed a head region. (PDF) [file pcbi.1010594.s005.pdf]

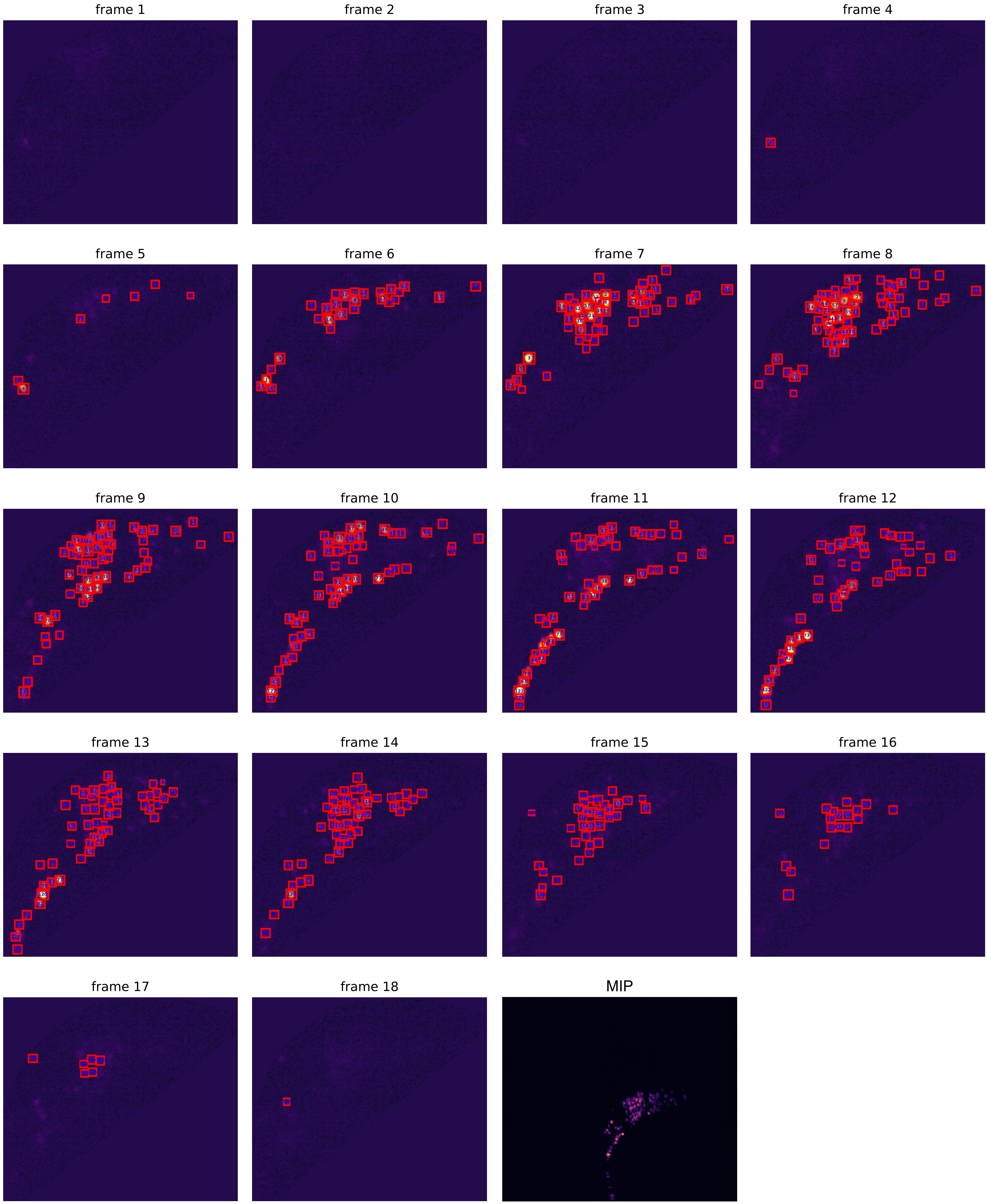

frame 1
frame 2
frame 3
frame 4
frame 5
frame 6
frame 7
frame 8
frame 9
frame 10
frame 11
frame 12
frame 13
frame 14
frame 15
frame 16
frame 17
frame 18
MIP
