# Supplementary material for: Rapid detection and recognition of whole brain activity in a freely behaving Caenorhabditis elegans
Source: PLoS Comput Biol. 2022 Oct 10;18(10):e1010594. doi: 10.1371/journal.pcbi.1010594 (PMC9584436; doi:10.1371/journal.pcbi.1010594)
Supplement: Attachment — Submitted filename: Response letter.pdf [file pcbi.1010594.s006.pdf]

Dear Blake and Daniele,

We are delighted to submit our revision, entitled "Rapid detection and recognition of whole brain activity in a freely behaving *Caenorhabditis elegans*" for your consideration.

We are grateful to all *three* reviewers for their thoughtful suggestions and valuable criticisms. To better evaluate and compare our recognition algorithm with other state-of-the-art methods, we included new experiments tested on the same benchmark (Table 4 and Table 5). We substantially rewrote and restructured the Methods and the Results section to incorporate our new results as well as to further improve the readability of the paper.

Several major changes have been made in the revision to address reviewers' primary concerns.

- We divided the neuron recognition task into *tracking within an animal*, and *tracking across animals.* Tracking within an animal is the main focus in this paper: a small number of volumes recorded from an animal are used for training, while the remaining volumes from the *same* animal are used for inference. Our few-shot learning method is particularly suitable for tracking neurons over a long recording, demonstrated by new experiments tested on NeRVE (~ 1400 volumes) and our own new dataset C1 (~ 350 volumes). Our recognition algorithm features short training time, rapid inference speed and high accuracy (88% with 30 training volumes on C1, 84% with 80 training volumes on NeRVE). A comparison between different methods under the same benchmark was shown in Table 4.

- We reported the performance of our method on *tracking across animals*, namely the inference was carried out on different animals. Two scenarios were considered: different animals from an identical strain, or different strains collected by different labs. Our algorithm has a moderate accuracy (~ 66.7%) when tracking across different animals from the same strain. We also run fDNC on our dataset, highlighting the challenge of accurate inference across different datasets. A comparison between different methods under the same benchmark was shown in Table 4.

- We distinguished between digital ID and cell-type ID in the Method section and clarified that our current method is *not* suitable for recognizing cell-type IDs across animals. We thank Reviewer 1 for explicitly pointing this out and we now demonstrate this point by testing our method on two NeuroPAL benchmarks, Chaudhary et al., 2021 and Yu et al. 2021 (Table 5).

- We rewrote Stage 4, neuron recognition section in the Methods and Results section to better explain our algorithms and motivate our potential readers.

Below, we provide a detailed list of responses to each reviewer's comments.

Responses to Questions

Reviewer #1:

Wu and colleagues present a novel and innovative method called CenDer that uses a set of algorithms to detect neurons in freely behaving C. elegans and track them across video frames in 3 dimensional volumetric images. These particular challenges were recently addressed by two innovative methods that were published in 2021 eLife papers. One method called 3DeeCellTracker was published by Wen C and colleagues in the Kimura, Hillman and Nemoto labs. Another method called fDNC was published by Yu X and colleagues in the Leifer and Linderman labs, who focused only on the challenge of tracking neurons in moving animals. In this new manuscript, Wu and colleagues use a combination of artificial neural networks and embeddings to achieve high accuracy on the tasks, but it seems they ultimately underperform 3DeeCellTracker which remains the state of the art. All that being said, the innovations brought forth by CenDer in this manuscript provide a new perspective on the existing problem and novel ideas that might be combined with the previous works to further improve accuracy for the challenge of detecting and tracking neurons. I believe that PLoS Computational Biology is an ideal venue for this manuscript and that readers will appreciate the ideas and innovations that are introduced by the authors.

Thank you!

The manuscript is nearly done and I suggest mostly minor revisions but I have three concerns that should be addressed. My first concern is that some sections lack clarity. Given how complex some of the ideas are, the confusing parts of this manuscript should be rewritten for clarity. This will help researchers to reuse the algorithmic advances in future work to attack similar problems.

Thank you! We have made several major changes in the revision to improve clarity. First, we rewrite Step 4, neuron recognition section in the Methods. Second, we provide a conceptual introduction of the neuron detection and recognition algorithms in the results section to better motivate our readers.

My second concern is that the CenDer program is provided in several pieces that were written in different programming languages and requires a lot of work to use at the moment. Since this manuscript introduces an end to end solution for detecting and tracking neurons, the software should be made available in such a way that users can easily run it on prepackaged example

datasets and also use it for their own datasets. One way to do this is to build a jupyter notebook for it, and an alternative way is to compile it and add a simple user interface.

Thank you for the suggestions! We are now working towards building a google colab for our algorithms. We expect to make it publicly accessible *in early August 2022*.

Lastly the performance comparisons to 3DeeCellTracker and fDNC use their reported values instead of comparing them on the same benchmark. For this reason it's impossible to compare all three methods to determine which is best. A true head to head comparison on the same benchmark should be added so that readers can decide what solution to choose among the three main choices.

Thank you for the suggestions. In Table 4, we now compare our tracking performance with 3DeeCellTracker and fDNC under the same benchmark.

1. Abstract: C. elegans is not a "mollusc", the phylum is Nematoda. Same for Line 8.

Thanks and we have corrected it!

2. Line 32-40: references should be added and addressed for "Simultaneous recognition and segmentation of cells: application in C. elegans" by Qu L, et al, Bioinformatics 2011; "A probabilistic atlas for cell identification" Bubnis G, et al, arXiv 2019; and "Probabilistic Joint Segmentation and Labeling of C. elegans Neurons" by Nejatbakhsh A, et al MICCAI 2020.

Thanks for bringing to our attention these references. We have added new contents from Line 32-36 and Line 52-56 to introduce their work.

3. Line 42: "interference" should be "supervision and correction".

amended!

4. Line 53: in "CenDer", why is "Detection" capitalized but "neuron" and "recognition" are lowercase? I suggest capitalizing the remaining two words and rename it CeNDeR.

We have amended the name!

5. Introduction: 3DeeCellTracker and fDNC are the current state of the art programs for neuron recognition and tracking but they're not explicitly mentioned in the introduction. Readers deserve a better explanation of the state of the art programs available now, more information on the algorithms used by these programs, and a more explicit explanation of how CenDer improves upon or adds to the current state of the art work.

We have now provided a brief introduction of fDNC from Line 44-49, and 3DeeCellTracker from Line 26-32, and the distinct features of CeNDeR from Line 72-89.

6. Line 71 and 123: what is "F", Frame? Figure 1C uses (x,y,w,h,z). The authors should unify their notation and use "Z" in place of "F". Or D and d, for depth, in case they think Z will be confused for integers and f for functions.

corrected!

7. Fig 1D: the dashed colored lines are barely visible and should be made thicker.

corrected!

8. Line 84: is N a free parameter set by the user or is it fixed for the head? It looks like N was chosen from Table S5 but the authors should clarify this.

N is a hyperparameter (Table S1). If you choose another animal or dataset, N will be different. The old version Table S5 choosing 141 was to train a more accurate recognition model. Every animal will have a different number of neurons to track.

9. Algorithm 1, Require: how is the head contour determined? I see it's the largest component after dilation but this should be made much more explicit to the reader.

The head contour is defined as the largest connected component in a binary image. Now added to the text in Algorithm 1.

10. Algorithm 1, procedure 5: how is the anterior-posterior directionality of the head determined? The algorithm generates a vector but how is the anterior direction of the worm determined?

In Algorithm 1, we mentioned specifically: The ventral-dorsal axis divides the head into two regions; the region containing a greater number of points of interest defines the anterior direction.

11. Algorithm 1, procedure 6: how does the dorsal-ventral axis assignment work if the worm is twisted? It's also unclear how the perpendicular angle is set to ensure it lies in the dorsal-ventral as opposed to any other in this axis, for example aligning instead with the left-right axis or some other angle between dorsal-ventral and left-right axes. The central moment is only sufficient to capture the anterior-posterior vector but the worm is a 3-dimensional deformable and twistable tube.

Thanks for the thoughtful comments. Note that Algorithm 1 relies on the general observation that C. elegans crawls on its side, and it would fail to build an accurate coordinate system in extreme scenarios: (1) the head is twisting itself; (2) the head and body are touching each other during an Omega turn. These hard examples require more sophisticated algorithms and human corrections, and are beyond the scope of the current paper. We now make these points explicit in the paper.

12. Line 91: why is the dilation filter larger than erosion? Please be explicit as to why empirical choices were made.

To better construct a fully connected head region, we let the size of the dilation filter slightly bigger than the erosion filter. We have made this point explicit in the text.

13. Line 110: please add a link to the results section for the ANN and specify that the details of its training/test set and validation are provided in that section.

added.

14. Fig 3D: this panel is confusing, repetitive, and doesn't really illustrate this portion of the algorithm. Please show just one iteration of the ANN architecture, then illustrate how multiple ANN outputs are unified into single neuron detection. Since PLoS Computational Biology targets a broad computational audience, it would be best to imagine a naïve reader and then help them understand this process as best possible.

Thanks for the suggestion! Fig 3D has been updated

15. Line 150: the underlying assumption in the partition algorithm is that during successive frames in an image volume, neural vertices can be displaced due to worm motion within the XY plane, but not up and down in Z which could then lead to mis-partitioning and unification of multiple neurons into one very long neuron, or mis-partitioning one neuron into several smaller pieces. The authors should address this explicitly. One way to deal with this issue would be to impose an upper and lower constraint on neuron size that is learned from the dataset.

Thanks for your excellent points. We now elaborate upon this and add a new paragraph to the end of that section:

"Our partition algorithm relies on the observation that the displacement of the neuronal region R_k is dominated by worm motion in the X-Y plane. In our experiments, \textit{C. elegans} was sandwiched between a flat agar pad and a cover-slip, thereby restraining head movements in the Z direction. Nevertheless, large up and down movements could lead to incorrect partition of a single neuron and unification of multiple neurons. One way to amend this problem is to impose an upper and lower bound on the longitudinal neuronal size. We notice that there is a strong correlation between the cell nucleus size in the X-Y direction and in the Z direction, suggesting that size information can be directly learned from data."

16. Line 152: what algorithm was used to determine if pixel intensity displays multi-modality?

Thanks for raising this question. Strictly speaking, testing whether the intensity distribution is multi-modal requires a statistical test using maximum-likelihood curve-fitting followed by a likelihood ratio test. However, this procedure can be time-consuming. Instead, we designed

a quick and simple algorithm: If the mean intensity profile of neuronal regions *I(z)* exhibits a local minimum at z_0, we will divide the neuronal object into two at this section.

20. Stage 4, Neural Recognition section: this is a very difficult read and it's one of the more novel stages of the algorithm. As it doesn't provide a good overview of what's to come, the purpose of each part as it pertains to the whole isn't clear, and the transitions between each part aren't clear either. The authors would greatly benefit from having a naïve reader go through this section to help them in rewriting it to improve its clarity. This is also true of some of the previous sections, but to a lesser extent.

Thanks for your thoughtful and constructive criticisms! We now completely rewrite the Stage 4, Neuron Recognition section. (1) We add a short introduction at the beginning of this subsection. (2) We unfold dense paragraphs by better explaining the motivation, the algorithms and the mathematical formula, supplemented by additional figure panels.

17. Lines 163-180: this section is confusing and should be rewritten for clarity. What's missing is both an overview, and also the motivation behind the various computations that take place. I assume the Dirac delta is used to approximate each neuron as a point but this should be made explicit. Why do the authors switch to cylindrical coordinates, is this for the purpose of computing the cosine similarity in the ANN? Why is the data sectioned into 4 quadrants?

See Line 217-228 for a new explanation. We copy the sentences below:

"In practice, directly discretizing Eq. \ref{eq:pair correlation} generates a very sparse feature vector that is difficult for a neural network (see below) to learn. We also need to take into account the observation that neuronal distribution is anisotropic: cells are extensively distributed in the X-Y plane but are confined in a narrow axial range. To extract meaningful statistics from data, we find it advantageous to design the feature vector in the cylindrical coordinate $[\rho,\phi,z]$, and to integrate $g_i(\rho,\phi,z)$ over a larger volume. We therefore count the number of neuronal regions \textit{in between} two concentric cylinders, whose depth and radius are given by $(\mathfrak{z},l)$, and $(\mathfrak{z}-\Delta\mathfrak{z},l - \Delta l)$ respectively (Fig \ref{fig:rec_feas}A). To capture density variations in the azimuth and depth directions, we further divide the volume of integration into 4 territories, anatomically corresponding to ventral-dorsal and left-right sides. Such a division is empirical, reflecting the information coding and sparsity trade-off in the density feature engineering."

What happens when the head bends in opposite directions, across successive image volumes, such that some neurons are now closer to other sets of neurons and the KNN computation reallocates which neurons end up in which clusters?

Indeed, deep head bend and large brain deformation represent worst-case scenarios that our current algorithm does not perform well. This is corroborated by our new recognition results, see Fig. 8D and the text in the results section, where we explicitly pointed out:

“The top-1 recognition accuracy increases monotonically with the number of training volumes, reaching an average 88.25% with 30 randomly sampled training volumes (Fig \ref{fig:recogPerformance}C). Furthermore, the accuracy approaches 95.48% with 130 training volumes. Across the 200 remaining test volumes, the recognition accuracy fluctuates, at times when the performance drops below 60% (Fig \ref{fig:recogPerformance}D). Examining the MIP images suggests that these instances correspond to rapid and deep head bends, when dramatic deformation caused some neurons to be much closer to other sets of neurons, precluding an accurate inference since our recognition method relies on relative positional information to identify neurons.”

In the discussion section, we suggest new ways to improve the current algorithm, such as incorporating color information.

18. Lines 195-203: this section is also confusing. The authors state that several modifications are performed to improve the discriminability of the model. These modifications should be covered in greater depth. I assume they're all summarized as making space between the features in their embedding to make them more robust to a variety of noise in the feature vectors? It's unclear why these choices were made and how they remedy the issues presented by real world videos. The authors need to improve their explanation here.

We now completely rewrite this section, see Line 259-279, with additional figure panels for better motivation and illustration.

19. Line 188: what data are included in feature vector c, is it just the KNN values? This should be made explicit.

The inputs to the recognition network is each neuron's feature vector, built by concatenating neuronal density feature and K-nearest neighbors feature from a given volume, see Fig 6C for an illustration.

21. Line 218: both Toyoshima Y, et al BMC Biology 2020 and Yemini E, et al Cell 2021 show that neurons in C. elegans are positionally variable and thus require additional techniques, such as color, to identify cell types such as AWA. Assigning neuron cell-type ID's across worms, using just the cell positions, leads to a considerable amount of mislabeling errors. The authors should address this and limit their claims to tracking neurons within single animals across image volumes. Neuron IDs in this paper appear to be limited to enumerations of N_i where i=1, …, total neurons. The authors should be explicit in explaining that their method can be used with other techniques for neural ID but cannot perform the actual cell type ID itself.

Thanks for the suggestions. We added several paragraphs throughout the paper to clarify this point. First, we added a section that provides formal definitions of digital IDs, cell-type IDs, and distinguish between them, Line 298-306. Second, we explicitly pointed out that CeNDeR is not a method for recognizing cell-type IDs, see Line 467-476.

22. Line 231: as is the standard for such publications, the authors should provide the x, y, and z resolution for their microscope in microns. This becomes especially important later on when discussing neuronal regions in pixel "box" sizes, since these are expected to be sensitive to microscope resolution. In addition to this, the algorithm is likely limited to a minimum number of volumes per second below which it can no longer track neurons across frames. The authors should state the minimum volume rate needed for their algorithm to work.

We have added the resolution information in the Methods *imaging setup* section. Briefly, the lateral resolution measured is 0.30 microns per pixel, and the axial scanning step is 1.50 micron. Indeed, box size must be introduced in the context of spatial resolution, which is now included in Table 3.

With a 10 ms exposure time per frame, and ~18 image frames per volume, our volume rate is ~ 5 Hz. There are two ways to reduce the volume rate. (1) Keep the step size in Z, while increasing the inter-volume interval. (2) Reduce the step size in Z. Since both our training and inference procedures are sequence-independent, increasing the inter-volume interval is not critical for performance accuracy. The second scenario, however, would significantly impact Stage 3, 3D merging. On the one hand, a smaller step size may lead to a better axial resolution. On the other hand, it may be more difficult to merge different neuronal regions due to the rapid movements of the neuron. A trade-off between speed and resolution must be considered. Given that most neurons' nuclei have a diameter ~ 2 μm, we expect that our step size has hit the sweet spot.

23. Table 1: 80 total training volumes seems a bit low for training an ANN, I assume there are many neurons in each volume. The table should state how many neurons are present in each set of volumes to give the reader a better sense of whether sufficient observations were used to train the ANN.

Added in Table 1 and Table 2

24. Table 2: anchor box sizes are measured in pixels but the relevant measure is microns since different microscopes and objectives will result in images with different resolutions. The authors should provide box sizes in microns, in addition to their pixel measurements. The authors should also discuss the minimum axial and temporal resolutions required for their algorithm to work and whether they recommend downsampling high resolution images to improve their algorithmic speed and accuracy. This is especially relevant given their highly specific choices for anchor box and cropped image sizes, and choice of ANN training dataset, which are likely to depend strongly on volumetric resolution.

Thanks for the suggestions. We have added the box size in microns in Table 3 and discussed different resolutions in the *Discussion section*.

25. Lines 288-289: two thresholds, 0.4 and 0.2 are mentioned but seem arbitrary. The authors should clarify how these were chosen.

The two values gave the best performance in the validation dataset.

26. Line 307: it's not clear why the link to Table S3 is given. The authors should clarify that this supplemental table shows why the values of d=56 and s=16 were chosen.

We have reorganized the table for optimal hyperparameters (Table S1), and removed a few tables that detailed the grid search procedures. Values in Table S1 gave the best performance in the validation dataset.

27. Table 3: as mentioned earlier, cross animal neuron ID is not verifiable without a method to perform cell type ID. The authors should remove this claim or use datasets that permit cell type ID to verify their claims. The Toyoshima Y et al 2020 and Yemini E et al 2021 papers make their real animal datatsets public. Wen C et al, and Yu X et al, both in eLife 2021 use both synthetic and real animal datasets to quantify the performance of their 3DeeCellTracker and fDNC algorithms, and these datasets are also publicly accessible. The authors can use any of these datasets to validate claims of cross animal ID if they wish to include this claim. That said I don't see the claim of cross animal ID as necessary for this paper to be novel or of high impact, it is already very strong without this extra work.

28. Table 3, Accuracy: the comparison here uses reported accuracies from different papers using different benchmarks. It's not an appropriate comparison. All algorithms should be tested on a common benchmark so that they can be compared head to head to determine which is best on each task. The authors also fail to report the published top-3 accuracy of fDNC which is 91.3% but this will no longer matter when all algorithms are tested on a common benchmark dataset. The authors should compare CenDer to 3DeeCellTracker, fDNC, and CRF by Chaudhary S et al, eLife 2021. A benchmark dataset was already created in the 3DeeCellTracker paper. The 3DeeCellTracker benchmark reused datasets from Toyoshima Y et al 2016 and Nguyen JP et al 2017 papers, and then added its own dataset to these. So the standard exists to use the existing benchmark datasets. The authors can add their own benchmark dataset to the existing ones if they feel that their dataset adds information that is relevant to the comparison.

We respond to 27-28 together. As mentioned in the summary of the rebuttal letter, there are several major changes in the current revision. First, we distinguish recognizing consistent digital IDs across 4D imaging volumes (which is identical to tracking neurons) from recognizing cell-type IDs. Second, in Table 4, we summarize our tracking performance and compare them with other recognition methods under the same benchmark, including NeRVE, 3DeeCellTracker and fDNC. Third, we explicitly pointed out that CeNeR is not a method for recognizing cell-type IDs (Line 467-476). Nevertheless, we applied our inference

method to the NeuroPal datasets and made a side-by-side comparison with other methods such as CRF and fDNC under the same benchmark (Table 5).

29. I can't verify that the software and algorithms work because they require a combination of python, matlab, and even Nvidia GPUs. I believe many readers will be unable to use this paper's innovation for the very same reasons that I can't. PLoS Computational Biology policy states "For studies that describe software for end-user applications, deposition either in a repository that enables remote code execution or instructions for installing and using the software are a prerequisite." As is the code doesn't conform to this standard. Since the code is largely in python and the matlab parts can be compiled into an executable, the authors should create a jupyter notebook that executes their code, provide a tutorial and examples to use it in the notebook, and allow users to upload their own volumetric images to the notebook so they can test these with the software.

Thank you for the criticism! We have updated some jupyter notebooks on our CeNDeR GitHub repository, https://github.com/Wenlab/CeNDeR. (e.g. recognition : https://github.com/Wenlab/CeNDeR/blob/main/recognition.ipynb). We will keep updating this repository as well as the README file, expecting a fully functional model that can be tested on any personal computer in *early August* 2022.

Reviewer #2:

Wu et al. describe CenDer, an interesting and speedy addition to the suite of algorithms for tracking of C. elegans neurons in whole-brain or whole-animal calcium imaging. This is a challenging problem for a variety of reasons, and is a very important one to solve well in order to allow biological insight to be derived from such recordings. In brief--we can watch worms think, but if we cannot keep track of which neuron is which even within an animal, much less across animals, we cannot understand what we are seeing.

The manuscript is clearly written, has figures that helpfully illustrate less-intuitive parts of the algorithm (e.g. concentric images), and sets out the current state of the art amply while describing the advances made by this paper. The main advance is that the method achieves quite-good performance with very little computation time--other approaches are either fast, but so poor as to be unusable (though to be fair it is not clear that the accuracy of this method is good enough either), or of good quality but slow enough to pose a major computational bottleneck. This paper's method computes neuronal identities only 4x slower than acquisition speed, making it very unlikely to be a bottleneck in any real setting. The best competitor, 3DeeCellTracker is 100x slower than CenDer and 400x slower than acquisition, meaning that an experiment that takes just three minutes will require a whole day to compute. Although this can

be ameliorated somewhat by running on a large cluster or in the cloud, this emphasizes that speed is in practice a significant concern.

The authors do not, in this paper, present a final solution to the problem: though their method is fast and accurate, for some applications it is unlikely to be accurate enough. This manuscript should therefore be viewed both as a potentially useful tool and as a report on a promising direction that the authors or other groups could continue to pursue until accuracy is adequate. (The authors describe some potential improvements in the discussion.)

Thank you!

The biggest flaw in the results is that there does not appear to be any validation on a recording from an animal that has not been used as part of the training set. Instead, every animal is used to contribute to both training, test, and validation data sets by splitting the recording in time. While this is valuable, it is far from adequate. Even if it is too labor-intensive to annotate enough individual animals to get ground-truth for a reasonable sample size, showing at least one new animal is very important. Or, alternatively, the authors should at least retrain their network with two out of the three animals, and use the third annotated animal as the test set. The neuronal clustering appears promising--but this may be partly illusory due to the selection criterion for which neurons were even allowed to exist (i.e. only those appearing in all three--a retraining must be done WITHOUT excluding missing neurons from the held-out animal!). If I am mistaken and this never-trained-on-animal data does exist already, it should be highlighted much more, as this is how the tool would be actually used: people would generate new data sets, not annotated by humans, and would run the software to get their results. For the biologist, nothing else really matters. As a computational technique, even if it turns out that this doesn't work, the paper still has merit for its clever and speedy preprocessing and feature construction which provides an appropriate level of detail as input to the ML part of the analysis pipeline.

We thank you for the constructive criticisms! In the new results section, we have divided our neuron recognition into two tasks, Line 410-465. In the within-animal-tracking task, a small fraction of volumes collected from a single freely behaving animal were used for training, while the remaining volumes from the same animal were used for testing. In the second task, we tested the tracking performance of our trained recognition neural network on a different animal (either the same strain or different strains generated by a different lab). Finally, we compared our training time, inference speed, and accuracy with other state-of-the-art recognition methods under the same benchmark.

In addition to this one main concern--hopefully an easily addressed one--there are a variety of minor comments below.

Abstract: C. elegans are NOT molluscs! Their phylum is Nematoda!

corrected!

Author summary: it would be more grammatically correct to say, "the fluorescent change in and thereby..." (add in). Alternatively, the sentence could be restructured.

We have amended this sentence.

Introduction: C. elegans are still NOT molluscs!

corrected!

Methods, Stage 1: The tense is wrong in "We design an Automatic Pre-Processing (APP) algorithm". This happened in the past: "designed". The running of it can be stated in present tense (e.g. "to extract a head region and build a C. elegans coordinate system"..."use it to" is redundant and actually a bit confusing).

corrected!

Methods, Stage 2: Here especially, but also elsewhere, the authors do not do a good job distinguishing between the *training* of an ANN and the *running* of the trained ANN to produce a result. Here, where the authors are describing the processing pipeline, "training" is not what is happening--the ANN is already trained. Thus, "Next, an Artificial Neural Network (ANN) is trained..." is not the right thing to say. The authors should clearly separate the two tasks:

(1) We trained an ANN on such-and-so data to find the center and size of candidate regions.

(2) We use this trained ANN as part of the processing pipeline.

corrected and thanks for the suggestions!

Methods, Stage 3: It is very difficult to refer to the NeuroAlignment step due to the unexpected fancy calligraphic X as the first character (it's not a LaTeX standard that I'm familiar with, but looks similar to fonts I do know). It would be nice if the authors could just call it XNeuroAlignment or something like that, so that it's completely clear what the first letter is.

corrected!

Methods, Stage 4: The K-nearest neighbors feature section says "Here we ignore the size of a neuron object by treating it as a point in three dimensions." But this is what we've already been doing all throughout the Neuronal density feature section! That's the whole point of the delta function. I do also wonder about the formalism--while the integrals are technically correct, the entire problem is discretized and really is a point-set problem. Thus, I wonder if it might not be simpler to cast the problem as an intersection between the point coordinate set X = {x_R_j} and a set of concentric partial shells about a neuron i, which you can form by defining the partial volume V(i, h, l) and then computing X ^ (V(i, h, l) - V(i, h - dh, l - dl)). Anyway, treating neurons as points is not new by the time we hit K-nearest neighbors.

corrected and we thank you for the suggestions, and more specifically Eq. (5).

Methods, Imaging Setup: "inverted microscopy" should be "inverted microscope".

corrected!

Methods, Red fluorescence channel...: Using the Fraktur font R, typically used to denote the real part of a complex-valued variable, is a peculiar choice. I understand that R is already in use for region, but this is liable to be confusing to any mathematician. Perhaps use Q, a calligraphic R instead of the Fraktur (\Re) one, or something else. Every time I see it, I think that, for instance, neurons are complex-valued.

corrected!

Figure 7: Same thing about Fraktur R used as ratio.

corrected!

Results, ANN training: It would be nice if the authors say something about how long training takes, not just running the trained model. It seems like it ought not take very long, given the limited size of the data set, but I couldn't find any numbers. Also, in the predefined anchor box size line, 9x9, 7x7, and 11x11 sizes in quotes have close-quotes on both sides. Either they should be plain quotes (neither open nor closed), or they should be a proper open-closed pair. (Yes, I know LaTeX will tend to make this mistake at times.)

corrected! The training time for neuron detection is ~ 4.5 hours when using a 9x9 anchor box.

Figure 9: The colors of the different steps are too similar. Please make them more dramatically different so it's easy to tell at a glance what step is what. For instance, they could all be blue of different darknesses (substantially different!), with the preprocessing in green, if that is to stand out.

Thanks for the suggestion and this figure has been redrawn!

Reviewer #3:

Wu and colleagues present a complete analysis pipeline for extracting calcium signals from large neural population recordings of moving C. elegans. Their pipeline segments neurons, tracks neurons through time, extracts calcium signals, and identifies the correspondence of neurons across animals. There has been a recent flurry of work in the literature presenting computational methods to address different aspects of this problem, which speaks to the interest and need in the community for developing a robust, high performing pipeline that could be of general use to the field.

My primary concern with this work is that the ground-truth evaluation of performance, as currently presented, is not yet satisfactory. If the reported performance holds up under more rigorous assessment, then I think this work would be a very valuable contribution to the field.

Most critical concerns:

1) Tracking performance (within-animal) should be more meaningfully assessed. Currently, performance is evaluated on an extremely small test set of 5 or 10 consecutive volumes representing less than 1 or 2 seconds of animal movement at the reported volume rate. This is woefully insufficient to evaluate the algorithm's performance on a representative sampling of animal poses or movements. Evaluation on longer recordings will also help differentiate the performance of this algorithm from sequence-dependent methods (Lagache et al., 2021) that perform well on short times, but struggle with longer recordings.

Thank you for the suggestions! In the new results section, we tested our within-animal-tracking performance on two datasets *with longer recording*: CeNDeR C1 with a total 350 volumes, and the publicly available dataset NeRVE with a total ~ 1400 volumes. In the within-animal-tracking task, a small fraction of volumes collected from a single freely behaving animal are used for training, while the remaining volumes from the same animal are used for testing. With 30 training volumes, our inference accuracy on the remaining volumes is 88.25% for the CeNDeR C1 dataset; 84.10% for the NeRVE dataset with 80 training volumes, suggesting that our sequence-independent algorithm is doing an excellent job on a long recording.

2) The quality of ground truth used to evaluate across-animal neural ID performance is unsatisfactory. Other recent works (refs 19-21) have used human annotations of NeuroPAL strains to establish ground truth neural ID across animals. There a human relies on genetically encoded color labels in addition to position and morphology. In this work, it seems that ground truth is

determined by a human using only position and morphology. I am skeptical of a human's ability to accurately identify neurons across animals via position and morphology alone. More evidence is needed to show that the ground truth across-animal neural correspondence used here for evaluation is accurate.

We understand your concern. There are two points we need to emphasize and clarify.

- Digital IDs and cell-type IDs are different (see formal definitions in the revision, Line 298-306, as correctly pointed out by you below).
- Human annotation is used to allocate ***consistent*** digital IDs across an imaging sequence within a freely behaving animal; digital IDs across animals ***do not have to*** correspond to each other.

With the above-mentioned facts in mind, we need to distinguish two different ground truth neural IDs: digital ground truths that are used to evaluate tracking performance and cell-type ground truths that are used to recognize a neuron's biological identity.

Throughout the paper, we use digital ground truths to evaluate our tracking performance. In the revision, as suggested by Reviewer 2, we have also considered two different kinds of tracking tasks. In the within-animal-tracking task, a small fraction of volumes collected from a single freely behaving animal are used for training, while the remaining volumes from the same animal are used for testing. In the across-animal-tracking task, we tested the performance of our trained recognition neural network on a different animal (either the same strain or different strains generated by a different lab). The across-animal-tracking performance is lower than within-animal-tracking performance, due to worm-to-worm variability. In the *Discussion session*, we suggest new ways to improve our current method.

What about cell-type ground truths? As clarified in the revision and correctly pointed out by Reviewer 1, our algorithm by itself cannot be used to recognize cell-type IDs. We now specifically address this point in our revision:

“CeNDeR is \textit{not} a method for identifying cell-type identities (see \nameref{sec:ID definition} for a definition). Neurons with defined cell-type exhibit large positional variability across worms \cite{Toyoshima_yu_2020, NeuroPAL}, both in absolute positions along dorsal/ventral and anterior/posterior axis, and in pairwise distances between neurons (Fig 2 in \cite{Toyoshima_yu_2020}), a finding that is inconsistent with the central assumption behind our recognition algorithm. Indeed, when testing our recognition method on the NeuroPAL datasets (Table \ref{table:all_dataset_setup}), which contain volumes from different animals with annotated cell-type IDs, the top-1 inference accuracy is merely 30% (Table \ref{table:rec_celltype_id}). Therefore, additional information, such as color \cite{NeuroPAL}, is required, and must be combined with our current method to allocate cell-type IDs.”

Other major substantive feedback:

- The work would be strengthened by evaluating performance on another dataset from a different group. It is becoming clear that imaging hardware, conditions and labeling approaches vary

across groups. If this work is to have the maximal impact on the field, it should show how well it can generalize to another C. elegans dataset. There are now at least a couple publicly accessible datasets in the literature. This also helps facilitate direct comparisons to other methods, because they can be evaluated on the same datasets.

Thanks! In Table 4, we summarize our tracking performance and compare them with other recognition methods under the same benchmark, including NeRVE, 3DeeCellTracker and fDNC.

- Please clarify when training must be performed, and all the instances where human input is required. For example, does the algorithm need to be trained anew for each worm? For each imaging setup? For each age of animal used or each strain? Make this information prominent in the text.

In the Discussion section, we now recommend how to use our recognition method to achieve the best performance:

- Annotate carefully a small number of imaging volumes that cover different head postures among your recorded 4D imaging dataset.
- Train the recognition neural network from scratch.
- Perform inferences on the remaining imaging volumes.

- Clarify how performance of this method is compared to competing methods in Table 3. Did the authors re-run those algorithms on a common dataset? If not, where did the values listed come from? If they came from the literature, then please double check. Some of the numbers listed do not match what I found in the literature.

These have been revised in the new Table 4 and Table 5.

- Neuron detection superficially appears to have some similarities to 3DeeCellTracker (Wen et al., 2020). Please expand on similarities and differences.

Ours is different from 3DeeCellTracker. 3DeeCellTracker uses extensive image registration and segmentation algorithms to detect neuronal objects, while ours does not involve any image segmentation. We strive to minimize pixel/voxel-wise computations in order to maximize detection speed.

Textual, visualization and some minor concerns:

- The detailed description of the pipeline can be hard to follow and reads more like a recipe. It would benefit from more high-level description to help guide the reader along and to better motivate each algorithm or implementation detail.

In the revision, we now include a summary at the beginning of the results section, aiming to conceptualize our algorithms at each step and how they might connect with each other.

- Figure 9 is hard to decipher because all the colors are similar and I cannot tell which bar is which. Please remedy.

corrected!

- Terminology is sometimes confusing. Please clarify when cell ID means ID across animals (e.g. the name in the connectome) vs the local identity within a worm.

Thank you for pointing this out! We include formal definitions of digital ID, cell-type ID in the Methods section, and refer to them when necessary in the main text.

- C. elegans come from phylum Nematoda not Mollusca . Remove all references to molluscan and mollusc.

corrected!

- Some language is odd. “Gadgets” in “Despite the presence of modern gadgets” is wrong.

corrected!

- 295: "A model was saved if it's performance has best" Please clarify. Does this refer to initializations of the parameters? Which are the different models?

During training, we computed the performance accuracy for the validation dataset in every epoch. If the accuracy of the current epoch was higher than a previous model with the best accuracy, we will save the new model. By the end of training, the model with the highest validation accuracy was found.
