# Supplementary material for: Rapid detection and recognition of whole brain activity in a freely behaving Caenorhabditis elegans
Source: PLoS Comput Biol. 2022 Oct 10;18(10):e1010594. doi: 10.1371/journal.pcbi.1010594 (PMC9584436; doi:10.1371/journal.pcbi.1010594)
Supplement: Attachment — Submitted filename: Reponse letter II.pdf [file pcbi.1010594.s007.pdf]

Dear Blake,

We are delighted to submit a second revision of our manuscript.

Below are the detailed responses to our reviewers.

Thank you!

Best,

Quan

Reviewer 2

(1) Line 6--"dynamic brain" should be "brain dynamics"

(2) Line 112--"filter slightly bigger" should be "filter be slightly bigger"

(3) Line 125--"detecting" should be "detect"

(4) Lines 301 & 302--"It is the consistency of a number across imaging volumes within an animal that allows the extraction" is grammatically correct but slightly awkward; the same point might be conveyed more comfortably with "Because it is consistent across imaging volumes within an animal, digital ID allows the extraction"

(5) Line 306--"have correspondence" might be clearer as "have a fixed correspondence across animals".

(6) Line 325--"thus have a" should be "thus has a"

(7) Line 431--"Noticeably" should probably be "Notably". (The clusters are noticeable, but "Notably" is the usual way to phrase such things.)

(8) Line 439--"at times when" should be "including a few cases where" (or something like that--it is important to point out that these are rare cases, which "at times when" doesn't do, in addition to suggesting that fluctuation is limited to the cases where it drops below 60%, which isn't really the case...the other fluctuations are just smaller).

(9) Line 442--"neurons, precluding an" should be "neurons. This precluded" (to help break up the overly-long sort-of-run-on sentence).

Thank you very much for carefully reading our manuscript! We have amended the text based on your suggestions.

Reviewer 3

Important clarifications needed in the main text:

Most importantly, the language about “within-animal” vs “across-animal” and “digital ID”, vs “cell ID” remains confusing despite the authors efforts to clarify in the text. For example, when I look at Table 4 I am inclined to think that “across-animal” tracking means tracking cell IDs across animals. But instead it refers to tracking digital neuron IDs within an animal, just using *training* from across animals. Please further clarify to the reader so this is less confusing. For example, language such as “within-animal tracking, via across-animal training” seems more accurate and clear.

Thank you! We now change the task names (with bold font) in the Results section as well as in Table 4.

Tracking within an animal becomes **Tracking neurons within an animal**

Tracking across animals becomes **Tracking neurons in a different animal with a pretrained model**

Similarly the definition of “digital ID” and “cell ID” (lines 298) and a mention that CeNDeR is only for “digital ID”s (line 471) should be made very early on in the paper and not a dozen pages in, so that a reader can quickly and accurately understand the scope of this work. For example around line 51 the authors should say something to the effective of “matching cell-type identities across animals is beyond the scope of the present work”.

In the new line 56-58, we now add: In this paper, we restrict ourselves to matching neuronal digital identities within an animal; matching cell-type identities across animals is beyond the scope of the present work.

Also in the author summary, the word “recognizes” in “detecting and recognizing most of the neurons” also seems to incorrectly convey give the impression that the algorithm finds cell IDs as opposed to digital neuron IDs. Please clarify in these areas too.

We now change recognizing to tracking.

Additionally, it it is important to explicitly remind the reader in the Discussion section about the non-trivial amount of user input required (30 frames) as a current challenge of the approach.

Line 524, we add: Human annotation of 30 volumes may still be a non-trivial amount of user input.

Minor:

-I recommend plotting 8C and 8E on a single plot for easier comparison

We decided to separate 8C and 8E for two reasons. (1) Each one has its own corresponding panel, 8D and 8F respectively. (2) The maximum number of training volumes, and therefore the scale in the C and E are different.

I think Figure 9CD would be more useful if it was combined so that the two algorithms could be compared on the same dataset

The old Figure 9 BCD has now been combined into a single panel 9B.
